# Who is at higher risk of hypertension? Socioeconomic status differences in blood pressure among Polish adolescents: a population-based ADOPOLNOR study

**DOI:** 10.1007/s00431-015-2554-0

**Published:** 2015-05-09

**Authors:** Maria Kaczmarek, Barbara Stawińska-Witoszyńska, Alicja Krzyżaniak, Małgorzata Krzywińska-Wiewiorowska, Aldona Siwińska

**Affiliations:** Department of Human Biological Development, Institute of Anthropology, Faculty of Biology, Adam Mickiewicz University in Poznań, Umultowska 89, 61-614 Poznań, Poland; Department of Epidemiology, Chair of Social Medicine, Poznań University of Medical Sciences, Poznań, Poland; Department of Pediatric Cardiology and Nephrology, Poznań University of Medical Sciences, Poznań, Poland

**Keywords:** Adolescence, Systolic blood pressure, Diastolic blood pressure, Place of residence, Parental education and occupation, Income adequacy, Family size, BMI

## Abstract

In Poland, there is no data on parental socioeconomic status (SES) as a potent risk factor in adolescent elevated blood pressure, although social differences in somatic growth and maturation of children and adolescents have been recorded since the 1980s. This study aimed to evaluate the association between parental SES and blood pressure levels of their adolescent offspring. A cross-sectional survey was carried out between 2009 and 2010 on a sample of 4941 students (2451 boys and 2490 girls) aged 10–18, participants in the ADOPOLNOR study. The depended outcome variable was the level of blood pressure (optimal, pre- and hypertension) and explanatory variables included place of residence and indicators of parental SES: family size, parental educational attainments and occupation status, income adequacy and family wealth. The final selected model of the multiple multinomial logistic regression analysis (MLRA) with backward elimination procedure revealed the multifactorial dependency of blood pressure levels on maternal educational attainment, paternal occupation and income adequacy interrelated to urbanization category of the place of residence after controlling for family history of hypertension, an adolescent’s sex, age and weight status. Consistent rural-to-urban and socioeconomic gradients were found in prevalence of elevated blood pressure, which increased with continuous lines from large cities through small- to medium-sized cities to villages and from high-SES to low-SES familial environments. The adjusted likelihood of developing systolic and diastolic hypertension decreased with each step increase in maternal educational attainment and increased urbanization category. The likelihood of developing prehypertension decreased with increased urbanization category, maternal education, paternal employment status and income adequacy. Weight status appeared to be the strongest confounder of adolescent blood pressure level and, at the same time, a mediator between their blood pressure and parental SES.

*Conclusion*: The findings of the present study confirmed socioeconomic disparities in blood pressure levels among adolescents. This calls for regularly performed blood pressure assessment and monitoring in the adolescent population. It is recommended to focus on obesity prevention and socioeconomic health inequalities by further trying to improve living and working conditions in adverse rural environments.

**Table:** 

**What is known:** • *Socioeconomic gradient exists in adolescent blood pressure levels.* • *Adolescents from lower SES families are at greater risk of hypertension.*
**What is new:** • *Urbanization levels of residence area affect adolescent blood pressure by parental socioeconomic status.* • *Socioeconomic inequalities in adolescent hypertension may be modulated through effects of body weight.*

## Introduction

The rate of diagnosis and prevalence of hypertension (HTN) in children and adolescents appear to show a steady upward trend. Recent reports have shown that prevalence of primary hypertension in the under-18 population varies from 5 to 20 % worldwide [27, 38, 84] and from 5 to 12 % in Poland [58, 60, 76].

Persistent elevation of arterial blood pressure (BP) is an independent risk factor for myocardial infarction and heart failure, stroke and end-stage renal disease. Hypertension and specific morbid sequelae have emerged as leading causes of premature death among adults worldwide [1]. Although it is rare for the young to develop atherosclerotic cardiovascular disease (CVD), the cumulative long-term effects of high BP may have multiple acute and chronic complications. There is evidence that high BP in young age is associated with early markers of cardiovascular abnormalities such as left ventricular hypertrophy and atherosclerosis [3, 6, 28]. Comorbid conditions include obesity, high blood lipid levels, learning and attention problems and type 2 diabetes [2, 13]. However, the main complication of persistent high blood pressure in young age is its progress to adulthood, making it the greatest cardiovascular disease risk [4]. Several lines of evidence suggest that young people who enter adulthood with higher BP parameters are more likely than their normotensive peers to be affected with HTN and its morbidity sequelae [19, 36]. Thus, earlier stages of life seem to be critical to HTN [20].

Hypertension is a common condition of multifactorial determination. It is suggested that HTN develops from a complex interplay of genetic, developmental, environmental and behavioural factors [38, 57, 61]. Heredity is a predisposing factor; almost half of young people with primary HTN have a positive family history of this condition, but environmental and individual contextual conditions may also play an important role in the development of HTN [61, 67]. A number of factors have been identified as predictors of elevated blood pressure in children and adolescents. They include maternal, birth and early-life characteristics, such as maternal weight status (BMI), smoking during pregnancy, low birth weight, breast feeding duration and childhood obesity [65, 70, 84]. Life cycle approach indicates that adolescence and especially puberty is a critical stage for adult BP. Longitudinal data showed that during puberty, BP may increase more than before it [81]. Rate of change in BP is likely to be synchronized with rapid somatic growth and adolescent growth spurt in height and weight [81, 86]. Moreover, changes in stature during puberty are closely linked to Tanner staging for sexual maturation. Gonadal hormones with possibly a preponderant effect of testosterone may affect BP levels and emergence of BP sexual dimorphism well manifest in adulthood [17, 29]. Additionally, adolescence is marked by increasing involvement in health risk behaviours often continuing into adult life [32]. Unfavourable effects of sedentary lifestyle, lack of vigorous or moderate physical activity, obesity, lack of a proper nutritionally balanced diet, high salt intake, low potassium and low calcium intake, tobacco use, alcohol intake and high stress may increase the risk for the development of adolescent hypertension [62, 63]. Furthermore, many of these factors are additive, such as unhealthy diet, insufficient physical activity and obesity, and vary in their propensity to contribute to the elevation of BP.

The literature on the potential confounders and mediators of childhood and adolescent hypertension has emphasized the role family level of socioeconomic status (SES) plays in the development of this condition [15, 55]. The association between parental SES and their offspring’s health outcomes has been well established [5, 16, 25]. These studies suggest that children of low-SES families are likely to have worse health outcomes. They are at a higher risk of CVD, elevated BP, metabolic syndrome, greater BMI and other negative health outcomes [43, 45]. They are also more likely to engage in risk-for-health behaviours than their better-off peers [34, 52]. Unlike heredity, ethnicity and geographic location, parental SES and adolescents’ behavioural factors are potentially modifiable. Elucidating the pathways by which these factors influence BP levels and health consequences (ischemic heart disease, stroke and others) may help in understanding the health gaps between different social groups and in developing a public health programme to counteract the health inequality [41].

In Poland, there is no data available for parental SES as a potent risk factor in adolescent hypertension, although social differences in somatic growth, development and maturation have been recorded since the 1980s [8, 10, 9]. This study aimed to fill that knowledge gap by focusing on SES differences in blood pressure (BP) levels among Polish adolescents 10–18 years old. The specific aims for this study were (i) to calculate prevalence of elevated blood pressure in relation to selected indicators of parental SES and (ii) to establish relative importance of SES-related factors on the development of high blood pressure. The study hypothesis was that adolescents living in low-SES families might be at a higher relative risk of elevated BP than their better-off counterparts.

## Materials and methods

### Study design and sampling

A cross-sectional survey was carried out between February 2009 and September 2010 on a representative, randomly selected sample of adolescents, aged 10–18 years, participants in the ADOPOLNOR project, a transdisciplinary study on adolescent health and quality of life. It was an ethnically homogeneous group of students in grades 5 through 6 of primary school, 1 through 3 of junior secondary and 1 to 2 of senior secondary schools in the Wielkopolska province and its capital, the city of Poznań.

Sample size was calculated using the formula for quantitative variable and a single cross-sectional survey [66]. The number of selected subjects was 5400.

Sampling procedure was a stratified two-stage cluster sample design. For the first sampling stage, schools were sampled from the sampling frame provided by the Ministry of Education for the Wielkopolska province via the Poznań Board of Education. Sampling was stratified by rural and urban areas as provided by Rogacki [79] and Central Statistical Office of Poland 2008 (www.stat.gov.pl). In this way, 52 schools were selected. The second sampling stage consisted of the selection of classes from the target grade of each participating school. In this procedure, if the number of classes was more than one, the class was randomly selected (as, for example, one class out of every six). In most villages, however, the students were assigned to only one class of each year level group.

The study design and study protocol were approved by the Bioethics Commission of the Poznań University of Medical Sciences (Resolution no. 311/07) and the Poznań Board of Education (Resolution WAF-405/1/JM/07). The survey was carried out in compliance with principles outlined in the Helsinki Declaration and subsequent amendments [90]. Schools’ headmasters received an invitation letter and an information brochure about the research project. They approved the study protocol and gave permission to run the study in their schools. Furthermore, in collaboration with them, subjects’ parents were informed about the goals of the study and possibility of refusing the participation of their children in the study. Enrolled for the study were those students whose parents had given a written consent for them to participate. In addition, students who had attained the legal age for consent (16 years in Poland) gave assent for their participation in the study. Almost all parents (97.1 %) provided written informed consent for their children to participate in the ADOPOLNOR research project and 96.7 % of young people aged between 16 and 18 gave us their written consent to be participants of the study.

Complete data on parental characteristics (demographic, socioeconomic, behavioural) and adolescent characteristics at time of investigation (medical examination, anthropometry, arterial blood pressure and physical fitness) were obtained for 2451 male and 2490 female students, the total of 4941.

All examinations were performed in school nursery rooms during morning hours (up to noon). The study protocol included medical examination, anthropometric measurements, and parental and self-reported background data questionnaires. Detailed description of the ADOPOLNOR study is available elsewhere [51].

### General health status

Health status of each subject was assessed by general practitioners (GPs) during general medical examinations, via self-report and proxy reports from parents.

### Anthropometric measurements

Body height and weight were measured by well-trained researchers according to standard procedures [56]. The subject, wearing light gym exercise clothes and without shoes, was standing in an upright position with heels together, arms to the side, legs straight, shoulders relaxed and the head positioned in the Frankfurt plane. The height was measured with a portable Swiss-made Gneupel Precision Mechanics (GPM) anthropometer to the nearest 1 mm from the highest point on the midline vault (vertex) to the floor a subject was standing on. Body weight was measured to the nearest 0.1 kg on a calibrated electronic scale (Precision Health Scale). Then, BMI was calculated by taking a subject’s weight (kg) and dividing it by his/her height squared (m^2^). Following the IOTF recommendation, Cole’s cutoff values were used to determine the weight status [23, 24].

Chronological age was calculated in decimal values by subtracting the date of examination from the date of birth. The age groups were divided by years, defined in terms of the whole year; e.g., 10 years old group involved subjects between 10.00 and 10.99 years old.

### Blood pressure measurements

Blood pressure was measured by school nurses strictly following the guidelines of the Fourth Protocol of the American Working Group of High Blood Pressure in Children and Adolescents [74]. A fully calibrated TECH MED TM-Z mercury gauge sphygmomanometer with sets of exchangeable cuffs and a clinical stethoscope was used for all BP measurements.

Blood pressure, systolic and diastolic, was measured in duplicate on each of the three occasions separated by a 2-day interval. Measurements were taken on the right arm with the subjects sitting for at least a 5-min rest, and the average of the two measurements was the final result for the given day as it was suggested in the Seventh Report for adults [22]. The systolic and diastolic BP measurements corresponded to the reading on the sphygmomanometer at the first and fifth phases of the Korotkoff sounds, respectively. The scale on the sphygmomanometer was graduated in 2-mmHg divisions. The readings were made to the nearest millimeter Hg. Calculated intra-observer technical error (intra-TEM) equalled 1.3 mmHg and inter-observer technical error (inter-TEM) equalled 2.3 mmHg [59]. The BP classification was determined using the surveillance method. For each participant, the mean of measurements taken on three occasions was calculated. The values of mean systolic blood pressure (SBP) and diastolic blood pressure (DBP) were adjusted by sex, age and height percentile using current reference data for Polish children and adolescents [59]. Normal BP was defined as systolic and diastolic BP less than 90th percentile, prehypertension (high normal BP) was defined as an average systolic or diastolic BP of greater than or equal to 90th percentile but less than 95th percentile, and hypertension was defined as an average systolic or diastolic BP of greater than or equal to 95th percentile [39].

### Socioeconomic status

Socioeconomic status was assessed through a self-reported family wealth using the Family Affluence Scale II (FAS II) and reports from parents using the ADOPOLNOR-R survey instrument. The SES indicators used in the study were the place of residence categorized according to the urbanization level (village with population of less than 1000 inhabitants, mainly engaged in farm work and this work is a source of income, small- to medium-sized city with population of less than 100,000, large-sized city with a population of 100,000 or more) [79], paternal and maternal educational attainment (the number of years of schooling completed and equalled to educational level: less than 12 years = primary/vocational level, 12 years = secondary level, more than 12 years = third level) and occupation status, family size (number of children in family), family finance-related burden referred to as *income adequacy* indicative of the objective financial situation, dwelling conditions and others (rated as an ordinal measure of more than enough, just enough or not enough money to cover expenses each month reported by study participants’ parents).

The FAS II, a four-item measure of family wealth, provided by students, was reported by number of cars in family, asking if the respondent have one’s own bedroom, number of family’s vacation travels during the past 12 months and number of computers in the household. The FAS II total score could range from 0 to 9, with higher scores indicating higher level of family wealth. In the study, it was scored as a composite score and classified into three categories: low affluence (0–2), middle affluence (3–5) and high affluence (6–9).

### Data analysis

The outcome of interest was demographic and parental socioeconomic factors associated with BP status (0 = normotension, 1 = prehypertension and 2 = hypertension) in adolescent males and females after controlling for parental hypertension, sex, age and weight status. At first, multiple correspondence analysis (MCA) was used to determine whether the explanatory variables for the BP status were associated to each other and which of them might potentially operate in an additive way [85]. Crude associations of BP status and all potential covariate variables were evaluated individually using the chi-square Pearson test. Multiple multinomial logistic regression analyses (MLRA) were used to assess the association between BP status and the variables in question. The dependent outcome variable was a dichotomous variable of BP status. Two models were evaluated: model 1 involving normotensive vs. prehypertensive BP status and model 2 involving normotensive vs. hypertensive status after adjustment for all potentially confounding variables simultaneously. The odds ratio was used as a measure of association. A final explanatory model with a subset and relative odds ratio (OR) of the factors associated with BP status was obtained using a stepwise procedure with backward elimination and rejection criterion of the *p* value greater than 0.05.

Statistical analyses were performed using the STATISTICA 10.0 data analysis software system (StatSoft Inc. Tulsa, OK, USA). All significance tests comprised two-way determinations. A value of *p* < 0.05 was considered statistically significant.

## Results

The social background of the sample is shown in Table 1.

**Table 1:** Characteristics of study participants on family history of hypertension, weight status and indicators of parental SES

Variables	*N* = 4941
	*n* (%)
Family history of hypertension^a^	1018 (20.6)
Weight status—BMI (kg/m^2^)	
Underweight	534 (10.8)
Normal weight	3548 (71.8)
Overweight	697 (14.1)
Obesity	162 (3.3)
Place of residence	
Rural areas	1897 (38.3)
Urban areas <100,000 inhabitants	2021 (40.9)
Urban areas ≥100,000 inhabitants	1023 (20.8)
Paternal education	
<12 years (primary, vocational)	2658 (53.8)
12 years (secondary)	1603 (32.5)
>12 years (university degree or above)	680 (13.7)
Maternal education	
<12 years (primary, vocational)	1947 (39.4)
12 years (secondary)	2013 (40.7)
>12 years (university degree or above)	981 (19.9)
Paternal occupation	
Economically inactive^b^	180 (3.6)
UB/PTJ/pension/others	513 (10.4)
Employed—full-time job	2974 (60.2)
Own business	727 (14.7)
Farming	547 (11.1)
Maternal occupation	
Economically inactive	763 (15.4)
UB/PTJ/pension/others	521 (10.5)
Employed—full-time job	2849 (57.7)
Own business	327 (6.6)
Farming	481 (9.8)
Number of children in family	
1 child	752 (15.2)
2 children	2312 (46.8)
3 and more children	1877 (38.0)
Income adequacy^c^	
Not enough	574 (11.6)
Enough	1832 (37.1)
More than enough	2535 (51.3)
Family affluence (FAS II)^d^	
Low	672 (13.6)
Medium	2764 (55.9)
High	1505 (30.5)

*UB/PTJ/pension/others* unemployment benefits/part-time job/life annuity/all others

^a^First-degree family history of hypertension: maternal and/or paternal hypertension

^b^A category that includes people who voluntarily remain out of the active workforce, those raising a family at home and/or those who are unemployed

^c^An ordinal measure of more than enough, just enough or not enough money to cover expenses each month reported by study participants’ parents

^d^Family affluence evaluated by adolescent participants in the study

The majority of families were urban residents (61.7 %) of working parents (60.7 % for both parents combined), fathers having fewer than 12 years of schooling (53.8 %) and mothers with 12 years of schooling (40.7 %), without financial strain, i.e., with income adequacy (51.3 % with more than enough income). Family affluence level was self-rated by study participants and the majority of them (55.9 %) rated it as medium. Families with two children accounted for almost a half of the sample (46.8 %); three and more children (38.0 %) were next in order of frequency.

**Fig. 1:**
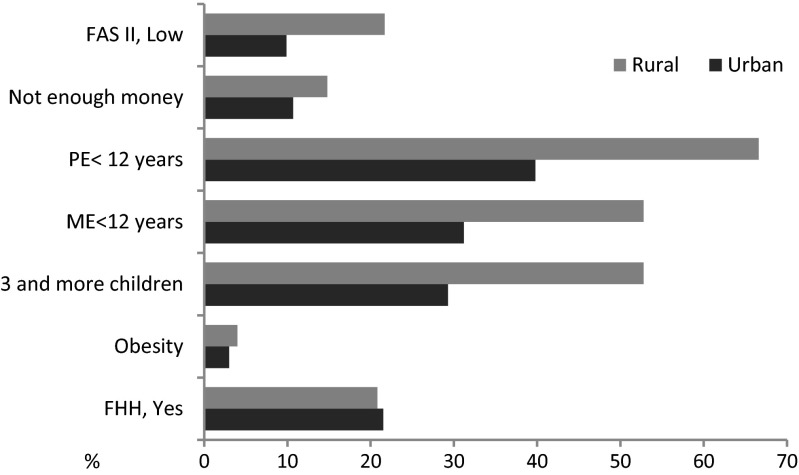
Rural-to-urban differences in selected indicators of parental socioeconomic status and family history of hypertension. For urban category small, medium and large cities combined. Abbreviations: *FAS II* Family Affluence Scale II, *PE* paternal education, *ME* maternal education, *FHH* family history of hypertension

Structural relationships among indicators of parental socioeconomic status—explanatory variables in the MCA (data not shown but available upon request from authors)—revealed that rural setting, low parental educational attainment, maternal economic inactivity, large families with three and more children, income inadequacy and low family wealth appeared to cluster closely together. Urban residence (<100,000 population) was associated with parental employment, income adequacy, medium family wealth and two children in family. One-child families were related to large city settings (≥100,000 population). The high affluent families markedly outlaid from other clusters indicating that the wealth was not associated with a specific setting or parental SES indicator. These associations conform to the pattern of additive nature of urbanization and parental SES factors. The rural-urban disparities in parental SES are shown in Fig. 1. Proportion of parents with low education level was significantly higher for rural areas as it was for income inadequacy and low family wealth (maternal education <12 years, 52.8 vs. 29.3 %; paternal education <12 years, 52.8 vs. 31.2 %; income inadequacy 14.8 vs. 10.7 %; low family wealth 21.7 vs. 9.9 % for rural and urban settings, respectively). There was also a significant rural vs. urban difference in the adolescent weight status. Prevalence of obese adolescents was higher in rural areas (4 vs. 3 %). Parental hypertension was equally distributed among inhabitants of rural and urban areas.

Table 2 shows the prevalence of BP status (SBP and DBP separately) in the study sample according to potential covariate variables. Crude associations between BP status and covariates are expressed in terms of chi-square test.

**Table 2:** Prevalence of prehypertension and hypertension among adolescent students by all factors involved in analysis

Variables	Blood pressure level							
	Normal		Pre-HTN		HTN		*p* value	
	SBP	DBP	SBP	DBP	SBP	DBP	SBP	DBP
Sex							0.009	0.612
Male	89.9	91.3	3.5	3.5	6.6	5.2		
Female	90.8	90.7	4.4	5.3	4.8	4.0		
Age (years)							0.042	0.006
10	90.5	92.9	3.9	3.4	5.6	3.7		
18	88.7	88.2	3.4	5.6	7.9	6.2		
Family history of hypertension							<0.001	0.026
Yes	84.5	88.7	5.7	5.6	9.8	5.7		
No	92.5	92.5	4.2	4.2	3.3	3.3		
Place of residence							<0.001	<0.001
Rural areas	85.3	86.0	5.7	6.8	9.0	7.2		
Urban <100,000 inhabitants	90.2	91.2	4.3	3.9	5.5	4.9		
Urban ≥100,000 inhabitants	92.2	94.3	3.4	3.0	4.4	2.7		
Paternal education							<0.001	<0.001
<12 years	88.5	89.1	4.5	5.1	7.0	5.8		
12 years	92.1	93.4	3.4	3.9	4.5	2.7		
>12 years	93.4	95.9	3.9	2.4	2.7	1.7		
Maternal education							0.002	<0.001
<12 years	88.9	87.7	3.9	6.2	7.2	6.1		
12 years	90.6	93.0	4.1	3.4	5.3	3.6		
>12 years	92.9	95.2	3.6	2.3	3.5	2.5		
Paternal occupation							0.005	0.003
Economically active^a^	91.3	92.7	6.0	4.3	2.7	3.0		
UB/PTJ/pension/others	89.1	90.0	7.6	5.8	3.3	4.2		
Economically inactive	86.1	87.4	8.2	7.3	5.7	5.3		
Maternal occupation							0.04	<0.001
Economically active^a^	94.0	94.1	4.0	3.1	2.0	2.8		
UB/PTJ/pension/others	89.1	87.9	6.6	5.1	4.3	7.0		
Economically inactive	86.2	84.3	7.9	8.9	5.9	6.8		
Number of children in family							0.05	0.004
1 child	91.9	93.8	3.4	2.8	4.7	3.4		
2 children	90.1	92.2	3.9	3.9	6.0	3.9		
3 and more	88.5	89.4	4.3	5.4	7.2	5.2		
Income adequacy							0.047	0.041
More than enough	92.5	92.5	4.1	4.0	3.4	3.5		
Enough	89.9	89.4	5.3	5.1	4.8	5.5		
Not enough	88.8	90.5	5.9	4.4	5.3	5.1		
Family affluence (FAS II)							0.532	0.151
High	89.4	91.9	6.7	4.4	3.9	3.7		
Low	89.4	89.8	7.1	5.0	3.5	5.2		
Medium	89.2	90.0	6.9	4.7	3.9	5.3		
Weight status—BMI (kg/m^2^)							<0.001	<0.001
Underweight	97.2	96.0	2.4	2.8	0.4	1.2		
Normal weight	92.3	92.9	4.4	3.8	3.3	3.3		
Overweight	83.0	82.9	9.1	7.7	7.9	9.4		
Obesity	58.2	72.1	15.1	14.3	26.7	13.6		

Values are in percentage

^a^Economically active category includes employed/own business/farming

In univariate analysis, the SBP levels were associated with all but the family affluence factor. Like systolic, diastolic BP was associated with all but sex and family affluence factors. There was a clear gradient in socioeconomic factors with a tendency of the disadvantage to locate in rural areas, parental low educational attainment, unemployment or farming and in income inadequacy. Higher prevalence of SBP as well as DBP HTN was found for participants with positive family history of hypertension (FHH) (SBP 9.8 vs. 3.3 % and DBP 5.7 vs. 3.3 % for yes and no, respectively) being at older age (7.9 vs. 5.6 % for 18 and 10 years for SBP and 6.2 vs. 3.7 % for DBP) and for male sex (SBP 6.6 vs. 4.8 %) with obese weight status (SBP 26.7 vs. 3.3 % and DBP 13.6 vs. 3.3 % for obese and normal weight status, respectively), living in rural than urban areas (SBP 9.0 vs. 4.4 % and DBP 7.2 vs. 2.7 % for rural and urban settings, respectively), having parents with low educational attainment (7.0 vs. 2.7 % for SBP and 5.8 vs. 1.7 % for DBP for fathers and 7.2 vs. 3.5 % for SBP and 6.1 vs. 2.5 % for DBP for mothers) and economically inactive (5.7 and 5.9 % for SBP and 5.3 and 6.8 % for DBP for fathers and mothers, respectively) with income inadequacy (5.3 % for SBP and 5.1 % for DBP) as compared to their better-off peers.

At multivariate level, only selected factors remained in their significance. The adjusted odds ratios for parental SES-related risk factors of pre-HTN and HTN after controlling for FHH, sex, age and weight status are presented in Table 3.

**Table 3:** Multiple/multinomial logistic regression analysis of most parsimonious set of factors affecting the likelihood of developing prehypertension and hypertension in adolescent students

Variable	Stepwise MLRA with backward elimination			
	Systolic blood pressure		Diastolic blood pressure	
	Prehypertension OR (95 % CI)	Hypertension OR (95 % CI)	Prehypertension OR (95 % CI)	Hypertension OR (95 % CI)
Sex				
Male (reference category)	1	1		
Female	1.24 (1.01; 1.54)	0.77 (0.59; 0.91)		
*p* value for trend	0.039	0.045		
Age (years)				
10 years (reference category)	1	1	1	1
18 years	1.23 (1.06; 2.01)	1.39 (1.09; 2.13)	2.30 (2.01; 2.97)	1.72 (1.05; 2.81)
*p* value for trend	0.046	0.038	0.005	0.029
Family history of hypertension				
No (reference category)	1	1	1	1
Yes	1.81 (1.31; 2.51)	1.72 (1.34; 2.20)	1.39 (1.01; 1.91)	1.43 (1.02; 1.99)
*p* value for trend	0.0003	<0.0001	0.039	0.036
Place of residence				
Rural areas (reference category)	1	1	1	1
Urban areas <100,000 inhabitants	0.82 (0.72; 0.94)	0.74 (0.66; 0.82)	0.64 (0.56; 0.73)	0.55 (0.48; 0.64)
Urban areas ≥100,000 inhabitants	0.56 (0.37; 0.82)	0.40 (0.29; 0.55)	0.26 (0.17; 0.39)	0.17 (0.11; 0.27)
*p* value for trend	0.004	<0.0001	<0.0001	<0.0001
Maternal education				
<12 years (reference category)	1	1	1	1
12 years	0.75 (0.69; 0.92)	0.73 (0.62; 0.86)	0.66 (0.54; 0.81)	0.60 (0.49; 0.74)
>12 years	0.60 (0.48; 0.91)	0.54 (0.39; 0.75)	0.44 (0.29; 0.66)	0.36 (0.24; 0.55)
*p* value for trend	0.002	0.0002	<0.0001	<0.0001
Paternal occupation				
Economically active (reference category)	1			
UB/PTJ/pension/others	1.24 (1.02; 1.50)			
Economically inactive	1.53 (1.04; 2.25)			
*p* value for trend	0.029			
Income adequacy				
More than enough (reference group)	1			
Enough	1.27 (1.12; 1.73)			
Not enough	1.40 (1.17; 1.94)			
*p* value for trend	0.019			
Weight status—BMI (kg/m^2^)				
Normal weight (reference category)	1	1	1	1
Overweight	2.9 (2.31; 3.64)	3.12 (2.63; 3.71)	1.89 (1.51; 2.38)	2.59 (2.10; 3.19)
Obesity	8.42 (5.33; 12.28)	9.75 (6.91; 13.75)	3.59 (2.28; 5.65)	6.75 (4.43; 10.15)
*p* value for trend	<0.0001	<0.0001	<0.0001	<0.0001

The likelihood of developing pre-HTN and HTN among adolescents from families with parental hypertension was almost twice as high as among those from families without FHH (OR = 1.81, 95 % CI 1.31; 2.51, *p*_trend_ = 0.0003 and OR = 1.72, 95 % CI 1.34; 2.20, *p*_trend_ < 0.0001) for SBP and 1.4 times as high for DBP (OR = 1.39, 95 % CI 1.01; 1.91, *p*_trend_ = 0.039 and OR = 1.43, 95 % CI 1.02; 1.99, *p*_trend_ = 0.036). Adolescent females were 1.2 times (OR = 1.24, 95 % CI 1.01; 1.54, *p*_trend_ = 0.039) more likely than males to develop systolic pre-HTN and 1.3 times less likely to develop systolic HTN (OR = 0.77; 95 % CI 0.59; 0.91, *p*_trend_ = 0.045). Adolescents at age 18 as compared to those at age 10 were 2.3 times (OR = 2.30, 95 % CI 2.01; 2.97, *p*_trend_ = 0.005) and almost 2 times (OR = 1.72, 95 % CI 1.05; 2.81, *p*_trend_ = 0.029) more likely to develop diastolic pre-HTN and HTN. They were 1.2 times (OR = 1.23, 95 % CI 1.06; 2.01, *p*_trend_ = 0.046) more likely to develop systolic pre-HTN and 1.4 times (OR = 1.39, 95 % CI 1.09; 2.13, *p*_trend_ = 0.038) more likely to develop systolic HTN.

Residents of large cities were almost twice less likely than their rural counterparts to develop systolic pre-HTN (OR = 0.56, 95 % CI 0.37; 0.82, *p*_trend_ = 0.004) and systolic HTN (OR = 0.40, 95 % CI 0.29; 0.55, *p*_trend_ < 0.0001). They were almost 4 times less likely to develop diastolic pre-HTN (OR = 0.26, 95 % CI 0.17; 0.39, *p*_trend_ < 0.0001) and almost 5 times to develop diastolic HTN (OR = 0.17, 95 % CI 0.11; 0.27, *p*_trend_ < 0.0001).

Compared to adolescents whose mothers had low level of education, peers having mothers with high/academic education level were 1.7 times less likely to develop systolic pre-HTN (OR = 0.60, 95 % CI 0.48; 0.91, *p*_trend_ = 0.002) and 1.8 times less likely to develop systolic HTN (OR = 0.54, 95 % CI 0.39; 0.75, *p*_trend_ = 0.0002). In addition, they were 2.3 times less likely to develop diastolic pre-HTN (OR = 0.44, 95 % CI 0.29; 0.66, *p*_trend_ < 0.0001) and 2.8 times less likely to develop diastolic HTN (OR = 0.36, 95 % CI 0.24; 0.55, *p*_trend_ < 0.0001).

Paternal occupation and income adequacy were two other factors associated with systolic pre-HTN. Using the employment status as reference category, the adjusted odds ratio for systolic pre-HTN risk from unemployment was OR = 1.53 (95 % CI 1.04; 2.25, *p*_trend_ = 0.029). Using the better-off financial situation (more than enough money) as a reference category, the adjusted odds ratio from income inadequacy for systolic pre-HTN risk was OR = 1.40 (95 % CI 1.17; 1.94, *p*_trend_ = 0.019).

Using the normal BMI for age as reference category, the adjusted odds ratios for systolic pre-HTN and HTN risks from obesity were OR = 8.42 (95 % CI 5.33; 12.28, *p*_trend_ < 0.0001) and OR = 9.75 (95 % CI 6.91; 13; 75, *p*_trend_ < 0.0001) whereas for diastolic pre-HTN and HTN were OR = 3.59 (95 % CI 2.28; 5.65, *p*_trend_ < 0.0001) and OR = 6.75 (95 % CI 4.43; 10.15, *p*_trend_ < 0.0001), respectively.

## Discussion

The present study provides the first data documenting social disparities in blood pressure levels among Polish adolescents. The findings revealed the multifactorial dependency of BP levels on geographic, i.e., rural or urban dwelling and SES-related familial influences at adolescence. The clustering structure of all factors involved in the analysis indicated that residential location might be operating through differential parental SES. The underlying pathways by which parental SES may influence their offspring BP levels include modifiable factors, such as the level of maternal education, status of paternal occupation and income interrelated to urbanization category of the place of residence after adjustment for FHH, subject’s sex, age and weight status. Consistent rural-to-urban and socioeconomic gradients were found in prevalence of elevated blood pressure, which increased with continuous lines from large cities through small- to medium-sized cities to villages and from high-SES to low-SES familial environments. Furthermore, the adjusted likelihood of developing HTN decreased with each step increase in maternal educational attainment, and pre-HTN decreased with increased maternal education, paternal employment status and income adequacy. The relationship between parental SES and BP levels as a gradient confirms persistence of social gradients that have been observed in Poland since the 1980s in other indicators of physical health [8, 10, 9].

Adverse consequences of low SES on BP levels and cardiovascular functions have been widely demonstrated in adults [77]. The findings of this study showed that social inequalities in BP levels manifest at adolescence. This is in line with the adolescent-emergent model (AEM) which states that relationships between SES and health outcomes are rather weak earlier in life but strengthen during adolescence when young people begin to be influenced by peers in their health behaviour [20, 30, 42]. Adolescence and, especially, puberty seems to be critical for the appearance of sexual dimorphism in BP which persists throughout adulthood. This finding is also in line with AEM and is most likely due to the activation of gonadal hormones with possibly a preponderant effect of testosterone involved during sexual maturation as well as acceleration in somatic growth during pubertal growth spurt [29, 81, 86]. The direct association of male sex with HTN and inverse association with pre-HTN found in our study need further analysis of data from longitudinal study.

As expected, elevated BP was independently associated with age. The likelihood of developing systolic pre-HTN and HTN increased twice with each year increase. Slightly weaker though significant association was observed between diastolic BP levels and age. Chronological age is a proxy for developmental trajectories. Its contribution varies in importance during each period prior to adulthood, so it does for BP level [18].

Not unexpectedly, our findings confirmed that parental HTN would be a major determinant of adolescent pre-HTN and HTN [54, 73]. The contribution of genetic determinants in developing high BP is accounted for 27 % of diastolic and 36 % of systolic BP [11]. Environmental exposures to permissive/adverse conditions via parental SES can be targeted in order to improve community and individual health. However, SES per se does not directly impact the physical status and physiological and functional capacity of growing individuals [12] neither health outcomes and so cannot be regarded as a treatable risk factor of elevated BP. There are several causal pathways that have been hypothesized for understanding the mechanisms that transfer geographical location and social and economic environment to health disparities at the community and individual levels. Rural communities are likely to be socially disadvantaged, facing job and neighbourhood strain, having low educational attainment, limited access to culture and the Internet and, in consequence, limited health literacy, which drive them to unhealthy behaviours ultimately resulting in chronic ill health, which, in turn, coupled with limited access to health care and low education level, may limit job opportunities. This is a vicious circle. Living in urban areas typically offers opportunities for better education, employment, better accessibility to health care services and adherence to medical treatment. On the other hand, rapid urban growth may generate numerous stressors resulting from population density, pollution, noise, unemployment and poverty [35, 53].

In Poland, disparities in somatic growth and selected health outcomes in the young age due to place of residence is a well-known phenomenon that has been reported by numerous studies. Despite inconsistencies as to the benefits of either environment, previous studies have shown that young people from urban areas are likely to be taller, thinner and earlier maturing as well as of better general health status as compared to their peers living in rural areas [8, 34, 48, 75]. Adverse effects of rural environment and its social structure on health outcomes found in our study are consistent with the results from the CBOS August 2013 report “Profile of the rural population” [78]. According to this report, the population living in rural areas showed that the better demographic situation characterized by positive demographic balance (the net population growth rate was higher in villages than in towns and cities—1.2 and 0.6 per 1000, in 2009, respectively) is accompanied by worse economic situation—a disposable per capita income of urban residents was twice as high as that of rural residents.

Importantly, maternal education, parental occupation, income adequacy and adolescent obesity remained significant when adjusted for all other relevant parent-related risk factors and place of residence. All these variables appear to act synergistically on adolescent BP levels via the acquisition of knowledge and skills that promote health associated with a higher level of schooling and the indirect effects of education on earnings and employment prospects [26, 87]. Wilson and colleagues, in their study on 76 black adolescents, revealed that adolescents who lived in poorer neighbourhoods had lower diastolic BPs if their mothers were more (vs. less) educated and their family had a higher (vs. lower) annual income [89]. The association between adolescent BP and maternal educational level has been demonstrated extensively [68, 83]. In our study, however, maternal occupational status interrelated with education and paternal occupation had no effect on blood pressure. Neither had family size. This finding is inconsistent with previous studies showing an independent effect of family size on offspring somatic growth [10, 48, 49]. At present study, parental educational attainment and earnings become more important for offspring BP than number of children at home.

The prevalence of elevated BP that has been reported in paediatrics recently varies substantially across countries [27, 31, 71]. The regional-wide variation in elevated BP prevalence is largely attributed to differences in geographic location, age range and methodology [39].

The overall prevalence of elevated BP was 6.6 % for pre-HTN and 8.9 % for HTN (SBP and/or DBP combined). These data indicate that the prevalence of systemic hypertension in the juvenile population in Poland has doubled over the last decade. Krzyżaniak and colleagues, in the national study of BP conducted in 2000 among Polish school children (7–19 years), reported the prevalence rate of HTN ∼4 % [58]. Similar figures, 4.9 % for HTN and 11.1 % for pre-HTN, were found in a large sample study of children and adolescents, aged 7 to 19 years in the city of Lodz, Poland [76].

This upward *trend* in HTN is attributed at least in part to the rapid increase in adolescent overweight and obesity [44, 50] and the high prevalence of sedentary behaviours, physical inactivity and unhealthy dietary habits [37]. According to recent data from the national survey in Poland, the prevalence of overweight and obesity in 6–19-year-old children and adolescents is 16.4 % (18.7 and 14.3 %, boys and girls, respectively) and underweight—12.0 % total (10.0 and 13.7 %, boys and girls, respectively) [44]. The findings of the present study showed similar figures: 16.8 % of overweight/obese adolescents in total sample (20.1 % for boys and 14.4 % for girls). Prevalence of obesity was found to be twice higher in boys (4.2 %) than in girls (2.4 %), and in rural (2.9 %) than in urban (1.9 %) residence areas. Although these figures are not top ranked among European adolescents (HBSC), predictions based on the worldwide trends suggest that it may change in the near future resulting in increasing risk of developing elevated BP and undesirable cardiovascular consequences [27, 72].

Brummett and colleagues revealed obesity and increased heart rate as key modifiable correlates of higher SBP and lower SES [15]. In the present study, weight status (BMI) appeared to be an independent most significant risk factor, suggesting its major role in mediating effects of parental SES on their offspring BP levels. In addition, it was found that higher parental SES was associated with lower BMI as it was claimed by Brummett and colleagues. There are many other studies identifying overweight and obesity as an independent significant factor of increased BP [21, 33, 37, 46, 80, 82].

This study is not without its limitations. A cross-sectional design makes it difficult to assess the direction and causality. This design, however, was methodologically appropriate for solving the research question, i.e., evaluating the association between BP levels (outcome variable) and exposures (parental SES, weight status) [69]. There is also a possibility that confounding factors operating earlier in life and not included to this analysis introduced bias into the study results. However, studies of the association between prenatal factors and offspring BP have yielded mixed results indicating direct or inverse associations and null results as well [7, 64]. An example of maternal smoking during pregnancy may prove that bias of present results, if any, can be neglected. Smoking during pregnancy is more common among women with low SES [47]; therefore, it was argued that the relation between smoking during pregnancy and offspring BP is due to SES confounding rather than a true intrauterine effect [14, 47]. Another bias may be produced by errors in recall of the exposure and possible outcome. It would have been useful to have longitudinal, prospective information. The reliability of self-reported data has widely been discussed in the literature and involved in the premises of this study [40]. Finally, SES indicators do not include disposable per capita income, but we share the opinion that income adequacy reflects more adequately the families’ purchasing power [88].

The strengths of this study include a population-based cohort study of healthy adolescents, the clustering structure of parental SES reflecting that of the general population (30), a multivariate approach and integration of multiple factors hypothesized as to be associated with the outcome variable—adolescent arterial BP level.

## Conclusions

The findings of the present study confirmed socioeconomic inequalities in blood pressure levels among adolescents. Young people living in rural areas are likely to be at a higher risk to develop elevated blood pressure than their better-off peers from urban areas.

Weight status appeared to be the strongest confounder of adolescent blood pressure level and, at the same time, a mediator between their blood pressure and parental SES. This calls for regularly performed BP assessment and monitoring in this population. Effective strategies aimed at reducing global CVD risk should focus on obesity prevention and socioeconomic health inequalities as early as at adolescence by further trying to improve living and working conditions in rural areas.

## Abbreviations

BMI: Body mass index
BP: Arterial blood pressure
CI: Confidence interval
DBP: Diastolic blood pressure
FAS II: Family Affluence Scale II
FHH: Family history of hypertension
GP: General practitioner
GPM: Gneupel Precision Mechanics
HTN: Hypertension
Intra-TEM: Intra-observer technical error
Inter-TEM: Inter-observer technical error
IOTF: International Obesity Task Force
MCA: Multiple correspondence analysis
MLRA: Multiple multinomial logistic regression analysis
OR: Odds ratio
Pre-HTN: Prehypertension
SBP: Systolic blood pressure
SES: Socioeconomic status
